# Commentary: Sodium levels and immunotherapy efficacy in mRCC patients with bone metastases: sub analysis of Meet-Uro 15 study

**DOI:** 10.3389/fimmu.2024.1476215

**Published:** 2024-11-07

**Authors:** Mehmet Fatih Ozbay

**Affiliations:** Department of Oncology, Kırşehir Training and Research Hospital, Kırşehir, Türkiye

**Keywords:** renal cell carcinoma, immunotherapy, prognostic factors, serum sodium levels, bone metastases, scoring systems

**To the Editor,**

I am writing in response to the recently published study titled “Sodium levels and immunotherapy efficacy in mRCC patients with bone metastases: sub analysis of Meet-Uro 15 study” (1). This work provides valuable insights into the prognostic significance of natremia in pretreated renal cell carcinoma (RCC) patients with bone metastases (BMs) receiving immunotherapy. However, I would like to address some concerns and suggestions for further analysis that could enhance the robustness of the findings.

Metastatic renal cell carcinoma (mRCC) is a challenging disease with a poor prognosis, particularly for patients with bone metastases. The effectiveness of immunotherapy in these patients has been limited, with response rates ranging between 20% and 40% (2). Identifying factors that may impact immunotherapy efficacy is crucial for improving outcomes in this patient population.

In addition to RCC, prognostic scoring systems have been widely used in other cancer types during the era of immunotherapy. For instance, in non-small cell lung cancer (NSCLC), the Lung Immune Prognostic Index (LIPI) has been developed, which combines the neutrophil-to-lymphocyte ratio (NLR) and lactate dehydrogenase (LDH) levels to predict outcomes in patients treated with immune checkpoint inhibitors. The use of LIPI has demonstrated that integrating inflammatory markers can significantly improve prognostic accuracy in patients receiving immunotherapy. This approach is similar to the proposed method for RCC patients, where serum sodium levels are incorporated. When looking at other cancer types, particularly NSCLC, it is evident that multivariable prognostic scoring systems provide valuable insights into patient outcomes and could potentially serve as a model for RCC as well (3).

Recent studies have suggested that hyponatremia, or low sodium levels, may be associated with worse prognosis in cancer patients, including those with metastatic renal cell carcinoma (mRCC) (4). Hyponatremia has been linked to increased inflammation, immune dysregulation, and altered tumor microenvironment, all of which could potentially influence the response to immunotherapy (5).

In particular, this study extends the findings of the previous work by Rebuzzi et al. (6), which evaluated metastatic renal cell carcinoma patients treated with nivolumab. In their study, Rebuzzi and colleagues developed a novel prognostic score focused on inflammatory indices and clinical factors. This current analysis further contributes to the literature by specifically evaluating patients with bone metastases, providing a more targeted assessment of their treatment responses.

## Analysis of hyponatremia across the entire patient cohort

The decision to focus exclusively on patients with bone metastases in this study is understandable, given the poor prognosis associated with BMs. However, a comprehensive analysis of hyponatremia across the entire cohort of RCC patients in the Meet-URO 15 study could provide more generalizable findings. By first analyzing hyponatremia’s prognostic value in the full patient population, the study could then offer a more targeted analysis within subgroups, such as those with BMs. This approach would clarify whether hyponatremia is a universal prognostic factor in RCC or if its significance is confined to specific patient subgroups (6).

By building on these findings, the current study has the potential to explore whether hyponatremia is an independent prognostic factor across the entire RCC cohort or whether its impact is modulated by specific conditions such as bone metastases. Furthermore, by integrating renal function into the analysis, as Attalla et al. suggested, it would be possible to assess whether the combined effect of hyponatremia and renal impairment contributes more significantly to poor outcomes. This could allow for a more nuanced understanding of the role sodium levels play in RCC prognosis, leading to more personalized and effective treatment strategies for patients based on their broader clinical profile (4).

## Consideration of renal function and serum creatinine levels

RCC patients frequently experience impaired renal function due to the nature of their disease and the treatments they undergo, such as nephrectomy and nephrotoxic medications. Since reduced renal function can contribute to hyponatremia, it is surprising that serum creatinine levels, an important indicator of renal function, were not considered in the analysis (7, 8). Ignoring this variable could confound the results, as hyponatremia might be more a reflection of impaired renal function rather than an independent prognostic factor. I suggest that future analyses should adjust for serum creatinine levels to more accurately isolate the impact of natremia on survival outcomes.

## The limitations of IMDC scoring in the era of immunotherapy

It is well known that the IMDC (International Metastatic RCC Database Consortium) score was developed and validated in the context of tyrosine kinase inhibitor (TKI) therapy, and its applicability to patients undergoing immunotherapy has been questioned. Recent publications have increasingly highlighted the importance of serum sodium levels in predicting outcomes for patients receiving immunotherapies (9–11). In light of these findings, it may be worth considering the development of a new risk scoring system that incorporates both serum sodium and creatinine levels. Such a scoring system could provide a more accurate prognosis for RCC patients undergoing immunotherapy, potentially guiding treatment decisions and improving patient outcomes.

## Conclusion

While the study makes a significant contribution to our understanding of prognostic factors in RCC patients with bone metastases, addressing the above concerns would further strengthen the findings. Specifically, incorporating renal function parameters and expanding the analysis to the full patient cohort would provide a more comprehensive assessment of hyponatremia’s role in RCC prognosis. Additionally, limitations of the IMDC scoring system stem from its focus on a limited set of parameters that may not fully capture the complexity of the tumor immune microenvironment. Factors such as the expression of immune checkpoint molecules, the presence and functionality of tumor-infiltrating lymphocytes, and the abundance of immunosuppressive cells within the tumor can all significantly impact the response to immunotherapy (12).

Moreover, the IMDC scoring system may not adequately address the dynamic changes in the tumor immune landscape that can occur during the course of treatment. As various immunotherapeutic approaches continue to emerge, the IMDC scoring system may become increasingly insufficient in accurately predicting and assessing the efficacy of these therapies.

Thank you for considering these suggestions. I look forward to further research in this area that builds on these important findings.

Sincerely,

Mehmet Fatih Ozbay

## References

[1] Catalano M Rebuzzi SE Maruzzo M De Giorgi U Buti S Galli L. Sodium levels and immunotherapy efficacy in mRCC patients with bone metastases: Sub analysis of Meet-URO 15 study. Front Immunol. (2024) https:1361010. doi: 10.3389/fimmu.2024.1361010 PMC11257879 39034992

[2] Mocellin S Wang E Marincola F. Cytokines and immune response in the tumor microenvironment. Immunotherapy. (2001) 24:392–407. doi: 10.1097/00002371-200109000-00002 11696695

[3] Mezquita L Auclin E Ferrara R Charrier M Caramella C Remon J. Association of the lung immune prognostic index with immune checkpoint inhibitor outcomes in patients with advanced non-small cell lung cancer. JAMA Oncol. (2018) 4:351–7. doi: 10.1001/jamaoncol.2017.4771 PMC5885829 29327044

[4] Penttilä P Bono P Peltola K Donskov F. Hyponatremia associates with poor outcome in metastatic renal cell carcinoma patients treated with everolimus: prognostic impact. Acta Oncologica. (2018) 57:1580–5. doi: 10.1080/0284186x.2018.1477256 29863419

[5] Ucà G Mariani L Vullo SL Galli G Berardi R Nicola MD. Weighing the prognostic role of hyponatremia in hospitalized patients with metastatic solid tumors: the HYPNOSIS study. Sci Rep. (2019) 9:1–10. doi: 10.1038/s41598-019-49601-3 PMC6736887 31506579

[6] Rebuzzi SE Signori A Banna GL De Giorgi U Pedrazzoli P Sbrana A. Inflammatory indices and clinical factors in metastatic renal cell carcinoma patients treated with nivolumab: The development of a novel prognostic score (Meet-URO 15 study). Ther Adv Med Oncol. (2021) 13:1–13. doi: 10.1177/17588359211019642 PMC8135208 34046089

[7] Lee SW Baek SH Ahn SY Na KR Chae D Chin HJ. The effects of pre-existing hyponatremia and subsequent-developing acute kidney injury on in-hospital mortality: A retrospective cohort study. PloS One. (2016) 11:e0162990. doi: 10.1371/journal.pone.0162990 27622451 PMC5021268

[8] Lim L Tsai N Lin M Hwang D Lin HY Lee J. Hyponatremia is associated with fluid imbalance and adverse renal outcome in chronic kidney disease patients treated with diuretics. Sci Rep. (2016) 6:36817. doi: 10.1038/srep36817 27841359 PMC5108044

[9] Ng TMH Cao D Patel K Wong YM Prasad M Lou M. Association of hyponatremia to diuretic response and incidence of increased serum creatinine levels in hospitalized patients with acute decompensated heart failure. Cardiorenal Med. (2014) 4:333–42. doi: 10.1159/000360604 24942293

[10] Ernst MS Navani V Wells JC Donskov F Basappa NS Labaki C. Outcomes for international metastatic renal cell carcinoma database consortium prognostic groups in contemporary first-line combination therapies for metastatic renal cell carcinoma. Eur Urol. (2023) 84:109–16. doi: 10.1016/j.eururo.2023.01.001 36707357

[11] Karaoğlan BB Yekedüz E Yazgan SC Mocan EE Köksoy EB Yaşar HA. Impact of low sodium values on survival outcomes of patients with cancer receiving immune checkpoint inhibitors. Immunotherapy. (2024) 16:821–8. doi: 10.1080/1750743X.2024.2370231 PMC11457628 39016058

[12] Hargadon KM Johnson CE Williams CJ. Immune checkpoint blockade therapy for cancer: An overview of FDA-approved immune checkpoint inhibitors. International Immunopharmacology. (2018) 62:29–39. doi: 10.1016/j.intimp.2018.06.001 29990692

